# Dr. Yeats, on Hydrencephalus

**Published:** 1816-05

**Authors:** G. D. Yeats

**Affiliations:** King Street, St. James's Square


*To the Editors of the Medico-Chirurgical Journal & Review.*

GENTLEMEN,

I HAVE just read the account which the Reviewer has been pleased to give in the Medico-Chirurgical Journal, of my humble attempt to elucidate the intricate subject of Hydrencephalus, in my letter to Dr. Wall. In his judicious observations on the subject, he seems to ascribe to me some contradictory opinions which I do not entertain, and which has probably arisen from my remarks not having been sufficiently clearly expressed. He observes, page 310, of the Number for the present month, " Dr. Yeats says, he has made it an established rule of practice, to join purgatives with the calomel; but we do not see how this idea accords with his recommendation of employing calomel as an alterative." The passage in my letter runs thus, p. 48: " The intestines are generally very torpid after the operation of a dose of calomel, and will continue so under its use, until it accumulates in the bowels, and even then it frequently does not act; for this reason it is, that in the digestive derangement which takes place in children, preceding the attack in the head, I have always found it necessary, and made it an established rule of practice, to join other purgative ingredients with the calomel, to obviate this state

(a morbid one previously described) of the duodenum, and thus to prevent the bile, and its other contents, from remaining too long in its cavity."

The torpid state of the bowels, after the exhibition of calomel as a purgative, is an effect which I have very commonly observed, and which has been also noticed by Dr. Cheyne particularly on this subject. The alterative effects which it is wished to be produced by mercury, will not be impeded by its union with a purgative, so as to maintain a regular peristaltic action, which in this disease is remarkably the reverse. It was not my intention to use an expression which conveyed the idea, that the calomel was to be hurried through the intestines by being conjoined with very active purgatives. I can assure the intelligent Reviewer, from no confined circle of observation on this disease, that a good deal of mischief has been produced from the want of some evacuating ingredient with the calomel, in consequence of the irritation which has arisen, not only from the accumulation in, but from a torpid state of, the bowels. In this way it is, that foul and torpid bowels, with irritation in the digestive organs acting upon the brain, have terminated in dangerous and fatal hydrencephalus, which would not otherwise have become such, as I have more particularly instanced in the fatal cases so candidly stated by the late Dr. Warren of Taunton.—" I am inclined to believe, that a fatal practice has been sometimes adopted from an inattention to this circumstance, and that cases, which have been treated as water in the brain, by the use of mercury only, have died from the want of the bowels being cleared by proper purgatives, although the mercury shall have done every thing that could be expected from its use, and only its intemperate and unassisted employment has rendered it abortive; hence, upon dissection, the head, although previously greatly complained of, has been found free from disease, and the bowels loaded with bilious foulness" *Letter to Dr. Wall*, p. 48-9.—And the following quotation will further show the ideas I entertained on the combined effects of an alterative and purgative medicine. " In by far the majority of cases, it is very unsafe to trust to the occasional exhibition of a purgative only; it is necessary to check the forming morbid action of the organs by the combination of an alterative with the purgative, in such a way as that you shall not merely remove the accumulated diseased load from the intestines by several large evacuations at once." p. 37-8.—So much, indeed, has mercury come under censure (as I suspect, from being unassisted by purgatives), that Dr. Blackall, in his very excellent and valuable work on Dropsies, has stated his belief that hydrencephalus has sometimes been produced by it.

The next observation I request to make is on the great and indispensable necessity of local bleeding in all cases of local inflammation :—The action of the heart is by no means an indication of the activity in the extreme vessels; if it is trusted to, *res est fallacissima,* we shall very commonly be deceived, and adopt a practice which will disappoint our expectations.

I have observed, "that general bleeding must not supersede the necessity of local detraction of blood is evident from the consideration that the activity in the extreme vessels, giving rise to the effusion, is independent of the action of the heart. You may therefore reduce the strong action of the heart, without overcoming the specific action in the extreme branches, constituting inflammation there." And, indeed, so full of conviction is my mind on the subject, that I have made use of the following very strong expressions :—"You may bleed generally till the heart is killed, without destroying the local activity, except by the destruction of the whole system." P. 63,4. I should not have thought it necessary to have adverted to this circumstance, had not the ingenious Reviewer referred to Dr. Parry's Pathology, to shew the necessity of local bleeding, thus implying that I had overlooked this very important and essential point of practice; my letter too was published before Dr. Parry's work issued from the press, or his opinions known to me. With respect to tonics, the Reviewer has very judiciously observed, that the best tonic is the removal of the disease; and I believe, in very many instances of all diseases, that will be sufficient: but it often happens, that after morbid action is subdued, the healthy action will be very feeble and debilitated, so as to require excitement; or very often it will have a healthy, but deficient secretion of bile from torpid action of the liver; or frequently a healthy, but feeble gastric action, the gastric juice not being secreted in sufficient quantity to produce a complete solution of the food; or sometimes a torpidity of the intestinal canal from the same cause: hence, some exciting tonic medicine becomes necessary, to rouse more energetic action, in order that more abundant secretions may be produced for the purposes for which they are severally intended. I trust, the candid Editors will understand that I have made these remarks from no wish pertinaciously to adhere to any preconceived theoretic opinions, but to illustrate my meaning, and to assist, by dispassionate enquiry, in elucidating the truth; for which, periodical Journals are particularly useful.

G. D. YEATS, M. D.

King Street, St. James's Square,
April 3, 1816.

---

P. S. I may here express a wish that Mr. Johnson, who has (p. 267) given the dissection of a case of hydrencephalus, caused by a fall, had also favoured the public with an account of the appearances of the epigastric viscera, especially as the first morbid symptoms are derangements of the abdominal viscera. I have read with much satisfaction this gentleman's publication on the Diseases of Tropical Climates, and I should have noticed his ingenious observations on affections of the head, caused by difficult hepatic circulation, had I seen his book previous to the publication of my letter. So striking a coincidence of opinion respecting the effects of a disordered circulation of the liver was perfectly accidental, but certainly merited, and would have had an acknowledgement from me, had I read his book previous to my publication. The nature of the internal congestions produced by the cold fits of fever, had occurred to me from practical observations many years ago; but Mr. Johnson's priority of publication, ingenuity of remark, and extensiveness of application, would justly have claimed my attention, had I been acquainted with them.

PART

